# A Missense Mutation in the *MYBPH* Gene Is Associated With Abdominal Fat Traits in Meat-Type Chickens

**DOI:** 10.3389/fgene.2021.698163

**Published:** 2021-08-11

**Authors:** Priscila Anchieta Trevisoli, Gabriel Costa Monteiro Moreira, Clarissa Boschiero, Aline Silva Mello Cesar, Juliana Petrini, Gabriel Rodrigues Alves Margarido, Mônica Corrêa Ledur, Gerson Barreto Mourão, Dorian Garrick, Luiz Lehmann Coutinho

**Affiliations:** ^1^Animal Science Department, University of São Paulo (USP)/Luiz de Queiroz College of Agriculture (ESALQ), Piracicaba, Brazil; ^2^Agri-Food Industry, Food and Nutrition Department, University of São Paulo (USP)/Luiz de Queiroz College of Agriculture (ESALQ), Piracicaba, Brazil; ^3^Department of Genetics, University of São Paulo (USP)/Luiz de Queiroz College of Agriculture (ESALQ), Piracicaba, Brazil; ^4^Embrapa Suínos e Aves, Concórdia, Brazil; ^5^School of Agriculture, Massey University, Wellington, New Zealand

**Keywords:** predicted deleterious SNPs, MYBPH, abdominal fat, chicken, meat-type

## Abstract

Chicken is an important source of protein for human nutrition and a model system for growth and developmental biology. Although the genetic architecture of quantitative traits in meat-type chickens has been the subject of ongoing investigation, the identification of mutations associated with carcass traits of economic interest remains challenging. Therefore, our aim was to identify predicted deleterious mutation, which potentially affects protein function, and test if they were associated with carcass traits in chickens. For that, we performed a genome-wide association analysis (GWAS) for breast, thigh and drumstick traits in meat-type chickens and detected 19 unique quantitative trait loci (QTL). We then used: (1) the identified windows; (2) QTL for abdominal fat detected in a previous study with the same population and (3) previously obtained whole genome sequence data, to identify 18 predicted deleterious single nucleotide polymorphisms (SNPs) in those QTL for further association with breast, thigh, drumstick and abdominal fat traits. Using the additive model, a predicted deleterious SNP c.482C > T (SIFT score of 0.4) was associated (*p-value* < 0.05) with abdominal fat weight and percentage. This SNP is in the second exon of the *MYBPH* gene, and its allele frequency deviates from Hardy–Weinberg equilibrium. In conclusion, our study provides evidence that the c.482C > T SNP in the *MYBPH* gene is a putative causal mutation for fat deposition in meat-type chickens.

## Introduction

Chicken is an important source of protein for human nutrition and a model system for growth and developmental biology (Ellegren, 2005). The genome sequence of the Red Jungle Fowl (*Gallus gallus gallus*), considered the ancestor of the domestic chicken (*G. g. domesticus*) (Abplanalp, 1992; Dodgson et al., 2011), and completed in 2004 (Hillier et al., 2004) has allowed the development of new tools for genetc studies. High throughput sequencing of several breeding lines has identified millions of single nucleotide polymorphisms (SNPs) across the chicken genome (Rubin et al., 2010; Boschiero et al., 2018) and these led to the development of high-density SNP panels (Kranis et al., 2013). The most common and frequent DNA variants are SNPs, with a density of 13 SNPs/kb in a Brazilian meat-type chickenline (developed by Embrapa Swine and Poultry) (Boschiero et al., 2018).

High-density SNP panels were previously used in genome wide association studies (GWAS) to identify QTL for body weight (Gu et al., 2011), several measures of fatness (Sun et al., 2013; Moreira et al., 2018b), breast and leg muscle weights, wing weight (Xie et al., 2012), carcass and eviscerated weights (Liu et al., 2013). However, discovering the causative mutations underlying quantitative trait loci (QTL) remains challenging (Ahsan et al., 2013; Moreira et al., 2018a). Linkage disequilibrium between the genetic variant present on a SNP panel and the casual mutation allows QTL detection by genome-wide association study (GWAS), but fine-mapping studies are necessary to identify which sequence mutation within the QTL is the causative mutation responsible for the phenotype of interest. Combining statistical evidence from association studies with functional annotations of the genes or genetic variants, is a helpful approach to identify potential causal mutations (Spain and Barrett, 2015).

Single nucleotide polymorphisms can have a direct impact on coding or indirect impact on regulation of gene expression and thus affect traits of economic interest in animal models and livestock species (Roux et al., 2014). SNPs that occur in coding regions can be classified as missense when triplets code for different amino acids, synonymous when triplets code the same amino acid or nonsense when the mutation results in a premature stop codon. Missense SNPs can be predicted as deleterious or tolerated using the SIFT tool (Sorting Intolerant From Tolerant) (Ng and Henikoff, 2003). Changes at well-conserved positions tend to be deleterious because important amino acids are conserved across a protein family (Ng and Henikoff, 2002, 2003). When a SNP is predicted as a deleterious mutation, it means that a change in the amino acid sequence likely affects the protein structure and function (Ng and Henikoff, 2003; Kumar et al., 2009), and consequently, may alter one or more phenotypes. Previous studies have identified missense SNPs associated with body weight at hatch, semi-eviscerated carcass weight, eviscerated carcass weight, leg muscle weight and carcass weight (Wang et al., 2015), abdominal fat weight, body weight at different ages and body size traits (Han et al., 2012). Phenotype may also be affected by SNPs located on potentially neutral regions (Berulava and Horsthemke, 2010; Jo and Choi, 2015), as these may be regulatory.

Previous whole-genome resequencing studies performed in parental individuals from the meat-type TT chicken reference population studied herein identified several predicted deleterious SNPs (Moreira et al., 2018b). However, the potential role of predicted deleterious SNPs in the regulation of traits of economic interest is still unknown. In this study, we used a GWAS to identify regions associated with carcass traits in a meat-type population, then integrated whole genome sequence data to refine the list of candidate mutations, followed by targetted re-sequencing to identify predicted deleterious SNPs and association study to identify putative causal mutations.

## Materials and Methods

### Experimental Population

The TT Reference population used in this study was generated from the Brazilian TT broiler line, developed by the Embrapa Swine and Poultry National Research Center. This line has been under multiple-trait index selection since 1992, representing many generations, with the goals of increasing body weight and carcass yield, improving viability, fertility, hatchability, feed conversion, and reducing abdominal fat (Do Rosário et al., 2009). The TT Reference Population was developed from expansion of the TT selection line comprising crossing of 20 males with 92 females in five hatches, yielding 1,430 chickens (Da Cruz et al., 2015; Marchesi et al., 2017).

### Phenotype Measurement

Body weight at 42 days of age (BW42) was measured six hours after fasting, then chickens were euthanized by cervical dislocation followed by bleeding. Blood samples were collected for DNA extraction during bleeding. Feathers were mechanically removed following a hot water bath (60°C for 45 s) and carcass cuts representing breast weight (BTW), thigh weight (THW), drumstick weight (DRW) and abdominal fat weight (ABFW) were individually measured in grams. Drumstick yield (DR%), abdominal fat yield (ABF%), thigh yield (TH%) and breast weight yield (BT%) were estimated as a percentage of live body weight at 42 days of age. More details about the slaughter and phenotype measurements have been previously described (Venturini et al., 2014; Da Cruz et al., 2015).

### DNA Extraction and Genotyping

**FIGURE 1:**
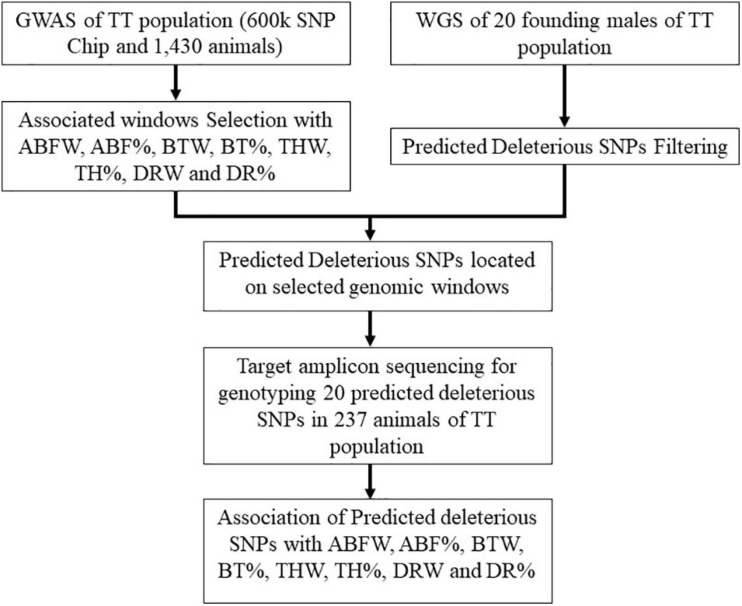
Workflow of genomic analysis performed.

Genomic DNA was extracted from 1,430 blood samples with the PureLink^®^ Genomic DNA kit (Invitrogen, Carlsbad, CA, United States) and quantified using Qubit^®^ 2.0 Fluorometer (Thermo Fisher Scientific, Waltham, MA, United States). For genotyping analyses, we used the 600 K Affymetrix Axiom^TM^ Genotyping Array (Affymetrix, Inc., Santa Clara, CA, United States). Sample filtering parameters were DishQC ≥ 0.82 performed with Axiom^TM^ Analysis Suite (Affymetrix^®^) software and sample call rate ≥ 90% via PLINK v.1.9 software (Purcell et al., 2007). For loci, the filter parameters of call rate ≥ 98% and minor allele frequency (MAF) ≥ 2% were used. We also excluded SNPs with significant deviations from Hardy–Weinberg equilibrium (HWE) (*p-value* < 0.000001), those located in the sex chromosomes, and those not annotated in the chicken assembly (Gallus_gallus-5.0, NCBI). More details about the genotyping and filtering steps are in Moreira et al. (2018b). Genomic analysis workflow is presented in Figure 1.

### Genome Wide Association Analysis

We used GenSel software for GWAS based on a Bayesian methodology approach for genomic prediction. Following Cesar et al. (2014) and Moreira et al. (2018b), a Bayes C model was used to estimate the genetic and residual variances for each trait that were used as priors in fitting a Bayes B model. The mathematical model used was:

$$y=X\,b+\sum_{j=1}^{k}a_{\text{j}}\,\beta_{\text{j}}\,\delta_{\text{j}}+e.$$

As described by Moreira et al. (2018b), **y** represents the vector of phenotypic values (BTW, BT%, THW, TH%, DRW and DR%); **X** is the incidence matrix for fixed effects; **b** is the vector of fixed effects; *k* is the number of SNP loci; **a_j_** is the column vector representing the SNP at locus *j* as a covariate, coded with the number of B alleles; β_j_ is the random substitution effect for locus *j* assumed to be normally distributed N (0, σ^2^_βj_) when **δj** = 1 but 0 when δj = 0, with δj being a random variable 0/1 indicating the absence (with probability π) or presence (with probability 1-π) of locus *j* in the model, and ***e*** is the residual associated with the analysis. Sex and hatch were included as fixed effects in the model and BW42 (slaughter age) as a fixed covariate for THW, BTW, ABFW and DRW.

We assumed π = 0.9970 in the BayesB models and obtained 41,000 Markov chain Monte Carlo (MCMC) samples with the first 1,000 samples being discarded. A map file was used to position the consecutive markers into 947 non-overlapping 1 Mb windows. So, for each window, the proportion of the genetic variance explained by the QTL was computed among individuals for every 100^th^ iteration of the MCMC chain based on the marker effects sampled in that iteration as performed by Schurink et al. (2012). The windows that had the marker with higher model frequency in the MCMC iterations had their effect predicted as mentioned by Van Goor et al. (2015). Each window is expected to explain 0.1054% of the genetic variance (100%/947) based on an infinitesimal model (Onteru et al., 2013; Van Goor et al., 2016). Were further considered as significant windows those that explained five times more variation than expected (0.53%) in an infinitesimal model, as used by Moreira et al. (2018b).

### SNP Selection and Custom Amplicon Design

Among the 20 founding males of the TT Reference population, 14 were resequenced by our group, resulting in approximately 13X sequencing coverage using a HiScanSQ (Illumina) sequencer and that produced a dataset of good quality SNPs we deposited in the EVA-EMBL database^1^. Further details about library preparation, sequencing and filtering are already published (Boschiero et al., 2018; Moreira et al., 2018b). Functional annotation of the SNPs was performed using VEP [Variant Effect Predictor, v.86, (McLaren et al., 2016)] and deleterious predictions were based on SIFT scores (Ng and Henikoff, 2003). To investigate the segregation of the predicted deleterious SNPs detected in the 14 founder males, we selected 237 descendents for targeted resequencing. These offspring represent 14 half-sib and 37 full-sib families. In each of the full-sib families, five to seven animals were re-sequenced. For the genotyping by sequencing methodology, a region of 150 bp centered around each predicted deleterious SNP was used to define a target region for amplicon design (DesignStudio online platform from Illumina).

This study investigated only predicted deleterious SNPs located within significant QTL regions associated with the carcass traits and abdominal fat traits (Moreira et al., 2018b), to narrow the causative mutation search. To avoid overlaps between the amplicons and maximize the number of regions sequenced, we used the Tagger tool in Haploview software (Barrett et al., 2005) to select one SNP per haplotype block of interest. Thus, if adjacent predicted deleterious SNPs exhibited r^2^ > 0.7, just one potential causative SNP was chosen to be genotyped by sequencing in the offspring generation.

### Target Sequencing

Genomic DNA of 237 offspring was extracted using the PureLink^®^ Genomic DNA kit (Invitrogen, Carlsbad, CA, United States) and then quantified using a Qubit^®^ 2.0 Fluorometer (Thermo Fisher Scientific, Waltham, MA, United States). DNA integrity was evaluated in 1% agarose gel. Library preparation was performed according to Truseq^®^ Custom Amplicon Low Input Kit Reference Guide (Illumina Technology). Libraries were quantified with quantitative real time PCR, using KAPA^®^ Library Quantification kit (KAPA Biosystem) and fragment size was estimated using either Bioanalyzer^®^ (Agilent Technologies) or a Fragment Analyzer (Advanced Analytical Technologies). Paired-end sequencing with a read length of 150 bp was performed on a MiniSeq^TM^ (Illumina Technology).

### Data Analyses, Variant Calling and Functional Annotation of Sequencing Data

We aligned the raw sequence reads against the chicken reference genome *Gallus_gallus*5.0 (NCBI) with BWA-MEM (v.0.7.15). For SNP calling, we used SAMtools v.1.3.1 (Li et al., 2009) with *mpileup* option (Li, 2011), and filtered based on mapping and base qualities (Phred) ≥ 20. The variant calling was performed after sequence reads from all 237 animals were pooled together. After the initial variant identification, we applied the following filtering options: INDEL removal, minor allele frequency (MAF) ≥ 0.05, SNP call rate ≥ 0.7, biallelic locus, sequencing depth ≥ 15 and Phred score quality ≥ 40.

After filtering, we annotated those SNPs that remained using the VEP tool version 91 (McLaren et al., 2016) available from the Ensembl v.91 website (Zerbino et al., 2018), where the SIFT score was predicted. We searched for gene ontology terms in Amigo 2 GO online server^2^. Deviations from Hardy–Weinberg equilibrium were calculated in Haploview software (Barrett et al., 2005) and Bonferroni multiple test correction was applied.

### Association Analysis

For association analysis, we tested 18 predicted deleterious SNPs located in QTL regions using the package lmerTest 3.1-0 (Kuznetsova et al., 2017) in R software (v. 3.5.2)^3^. To investigate the SNP effects, we fitted both additive and non-additive models for each trait (BTW, BT%, THW, TH%, DRW, DR%, ABFW, and AB%). As adopted by Edwards et al. (2015), for the additive models the SNPs were considered as continuous variables (0, 1, or 2 copies of a given SNP) and in the non-additive models the variable genotypes were considered as a factor (with three levels i.e. aa, Aa, AA). Sex and hatch were added in the model as fixed effects, and family (determined by the mother) was fitted as a random effect. For the carcass weight traits (BTW, THW, DRW and ABFW), BW42 was used as a fixed covariate. For each trait, all the SNPs located in the QTL associated with the respective trait were simultaneously fitted in the following model:

y=X⁢β+W⁢a+Z⁢u+e

Where ***y*** is the vector of observations for the measured phenotype; ***X*** is the incidence matrix relating the fixed effects for sex and hatch to ***y***; ***β*** is the vector of sex and hatch fixed effects; ***W*** is the genotype matrix for the predicted deleterious SNPs located in the associated QTL regions; and ***a*** is the vector of fixed effects for the SNPs. The matrix ***Z*** is an incidence matrix relating ***u*** to ***y***; ***u*** is the vector of length 41 representing the random effect of dam family; and ***e*** is the vector of residual effects. Associations were considered significant at comparison-wise *p-value* < 0.05.

## Results

### Phenotype Measures and Descriptive Statistics

Descriptive statistics such as number of animals, averages and standard errors from traits and animals utilized in GWAS are presented in Table 1. Summary statistics for all traits and animals used in genotyping and single SNP association analysis are presented in Table 2.

**TABLE 1:** Number of animals (N), mean, standard deviation (SD), minimum and maximum values for carcass traits from animals utilized for GWAS analysis in the TT Reference population.

**Trait^1^**	**N**	**Average**	**SD^2^**	**Minimum**	**Maximum**
BW42 (g)^3^	1,311	2,220.30	258.86	988	2,971.00
THW (g)	1,289	309.57	45.76	192	464.40
TH %	1,289	13.92	0.94	9.83	16.87
ABFW^3^	1,287	47.10	14.03	8.0	87.90
ABF%^3^	1,287	2.13	0.62	0.25	4.67
DRW (g)	1,281	205.38	31.11	128	274.00
DR%	1,281	9.23	0.62	7.25	12.14
BTW (g)	1,286	499.32	65.56	260	660.00
BT%	1,286	22.33	2.08	17.26	27.14

*^1^BW42, body weight at 42 days of age (in grams); THW, thigh weight (in grams); TH, thigh yield (%); DRW, drumstick weight (in grams); DR%, drumstick yield (%); BTW, breast weight (in grams); BT%, and breast yield (%). ^2^Standard deviation. ^3^Already published (see Moreira et al., 2018b).*

**TABLE 2:** Number of animals (N), mean, standard deviation (SD), minimum and maximum values for carcass traits from animals utilized for single SNP association analysis in the TT Reference population.

**Trait^1^**	**N**	**Average**	**SD^2^**	**Minimum**	**Maximum**
BW42	237	2,219.75	254.87	1,310.00	2,816.00
THW	237	203.75	30.27	110.80	277.80
TH%	237	9.16	0.63	7.26	11.64
ABFW	237	50.93	14.63	19.00	91.00
ABF%	237	2.29	0.61	1.01	4.25
DRW	237	30.01	44.63	161.60	419.20
DR%	237	13.80	0.89	11.81	16.35
BTW	237	495.39	61.93	260.00	660.00
BT%	237	22.32	1.31	18.28	26.51

*^1^BW42, body weight at 42 days of age (in grams); THW, thigh weight (in grams); TH, thigh yield (%); ABFW, abdominal fat weight (in grams); ABF%, abdominal fat yield (%); DRW, drumstick weight (in grams); DR%, drumstick yield (%); BTW, breast weight (in grams); BT%, and breast yield (%). ^2^Standard deviation.*

### Genome-Wide Association Analysis

We conducted a GWAS to identify genomic regions associated with traits of interest. Nineteen unique QTLs for THW, TH%, DRW, DR%, BTW, and BT%, were detected on GGA 1-4, 6, 12, 14, 17, 20, 22, 25, 26 and 28 (Table 3). The QTL explained from 0.54 to 3.2% of the genetic variance. The posterior probability of association (PPA) (Onteru et al., 2013) ranged from 0.69 to 0.99. Characterization of all 947 genomic windows analyzed is available in Supplementary Table 1. For ABFW and ABF%, nine unique QTL had been previously reported (see Moreira et al., 2018b).

**TABLE 3:** Significant genomic windows associated with THW, TH%, DRW, DR%, BTW, and BT% in the TT Reference population.

**Trait**	**GGA (Mb)^1^**	**SNP window**	**Number of SNPs/window**	**Proportion of genetic variance explained by the SNP window**	**PPA^2^**
		**(first – last position)^1^**			
THW	22 (4)	4,000,760 – 4,676,714	1035	0.54	0.95
TH%	2 (62)	62,001,908 – 62,998,786	310	0.62	0.69
	22 (4)	4,000,760 – 4,676,714	1035	0.57	0.97
DRW	1 (56)	56,000,162 – 56,998,456	358	0.71	0.80
	1 (166)	166,000,511 – 166,999,195	390	3.20	0.92
	2 (40)	40,007,952 – 40,999,399	305	0.56	0.72
	3 (30)	30,000,897 – 30,993,376	386	1.15	0.83
	4 (24)	24,000,906 – 24,996,950	321	0.61	0.78
	6 (8)	8,002,588 – 8,996,459	508	1.51	0.90
	28 (0)	24,369 – 999,295	883	0.80	0.99
DR%	1 (166)	166,000,511 – 166,999,195	390	2.79	0.93
	2 (9)	9,012,190 – 9,990,590	310	0.62	0.74
	3 (30)	30,000,897 – 30,993,376	386	1.39	0.85
	4 (24)	24,000,906 – 24,996,950	321	0.55	0.76
	6 (8)	8,002,588 – 8,996,459	508	0.99	0.90
	14 (3)	3,001,973 – 3,999,889	549	0.53	0.89
	20 (7)	7,000,004 – 7,998,579	475	0.54	0.86
	28 (0)	24,369 – 999,295	883	0.70	0.97
ABFW^3^	5 (38)	38,000,437 – 38,996,916	396	0.92	0.84
	10 (7)	7,000,336 – 7,998,549	592	0.58	0.93
	13 (3)	3,002,617 – 3,998,616	460	1.45	0.88
	20 (5)	5,000,651 – 5,999,452	492	0.94	0.88
	26 (1)	1,002,598 – 1,999,851	662	1.06	0.95
ABF%^3^	5 (38)	38,000,437 – 38,996,916	396	0.64	0.82
	10 (7)	7,000,336 – 7,998,549	592	0.61	0.90
	13 (3)	3,002,617 – 3,998,616	460	1.49	0.89
	26(1)	1,002,598 – 1,999,851	662	0.54	0.92
BTW	17 (7)	7,000,143 – 7,999,150	667	0.64	0.92
	25 (2)	2,001,192 – 2,887,176	512	0.81	0.88
	26 (2)	2,000,388 – 2,999,606	904	0.66	0.95
	26 (3)	3,000,141 – 3,998,650	998	0.53	0.96
BT%	4 (43)	43,001,322 – 43,998,491	364	0.61	0.80
	12 (5)	5,000,340 – 5,999,121	596	0.64	0.89
	12 (6)	6,000,683 – 6,997,857	550	0.74	0.87
	17 (7)	7,000,143 – 7,999,150	667	0.65	0.91
	25 (2)	2,001,192 – 2,887,176	512	0.60	0.84
	26 (2)	2,000,388 – 2,999,606	904	0.87	0.97
	26 (3)	3,000,141 – 3,998,650	998	0.86	0.98

*^1^Map position based on Gallus_gallus-5.0 NCBI assembly. ^2^Posterior probability of association (PPA) as reported by Onteru et al. (2013). ^3^Already published (see Moreira et al., 2018b). DRW, drumstick weight; DR%, drumstick yield; THW, thigh weight; TW%, thigh yield; BTW, breast weight; BT%, breast yield; ABFW, abdominal fat weight; ABF%, abdominal fat yield.*

Windows that explained a high proportion of genetic variance were selected for each carcass trait to further search for predicted deleterious SNPs. The genomic windows selected were: GGA 1 (166 Mb) for drumstick traits; GGA 22 (4 Mb) for thigh traits; GGA 26 (1 Mb) for abdominal fat traits [9]; GGA 25 (2 Mb) and GGA 26 (3 Mb) for breast traits.

### SNP Selection and Amplicon Design

We searched for predicted deleterious SNPs located within the QTLs to identify potential causative mutations. We scrutinized SNP data from whole-genome resequencing of 14 founding animals (TT line), approximately 11 million SNPs (Boschiero et al., 2018), concordant with the five selected GWAS windows. This recovered 89 predicted deleterious SNPs. After linkage disequilibrium pruning in the founder’s dataset, 18 uncorrelated predicted deleterious SNPs were selected for amplicon design and association analysis. One predicted deleterious SNP was tested for each of the following regions: GGA 1 (166 Mb) window associated with DRW and DR%, GGA 22 (4 Mb) associated with THW and TH% and GGA 25 (2 Mb) associated with BRW and BR%. For GGA 26 (3 Mb) associated with BRW and BR% and GGA 26 (1 Mb) associated with ABFW and ABF%, seven and eight predicted deleterious SNPs that were not in linkage disequilibrium were tested, respectively.

### Amplicon Sequencing, Variant Calling and Functional Annotation

Libraries sequenced using MiniSeq produced on average 298,791 raw reads per amplicon. The average overall mapping rate of the raw reads against the *Gallus_gallus*5.0 (NCBI) genome assembly was 99.74%. After variant calling, quality control and functional annotation, the 18 predicted deleterious SNPs were genotyped by sequencing with an average coverage of 7,000X.

Detailed information of the 18 predicted deleterious SNPs (genome position, SNP ID, located gene, allele and genotype frequencies, HWE test and SIFT score) is in Table 4. The 18 predicted deleterious SNPs were detected in the offspring, validating our whole genome sequence data and SNP calling. Two SNPs are novel so did not have *rs* IDs, and four SNPs were annotated in novel genes. Seven SNPs did not have any animal genotyped as homozygous for the alternative allele, and five SNPs exhibited significant deviation from HWE.

**TABLE 4:** Deleterious SNPs selected for the association analyses with carcass traits.

			**Tested**	**Gene**			**Allele**								**HW**	**SIFT**
**GGA**	**Position**	**SNP ID^1^**	**Trait^1^**	**Symbol**	**Ensembl Gene ID**	**R/A**	**Frequency**		**Genotype Frequency**						***p*-value**	**score**
									**HREF**		**HT**		**HALT**			
							**R**	**A**	**Freq.**	**N**	**Freq.**	**N**	**Freq.**	**N**		
1	166,014,604	rs739508259	DR	*VWA8*	ENSGALG00000016955	G/C	0.588	0.412	0.364	86	0.449	106	0.186	44	0.6262	0.01
22	4,589,985	rs314536739	TH	*ANXA4*	ENSGALG00000038783	C/T	0.738	0.262	0.531	126	0.413	98	0.054	13	0.4782	0.02
25	2,264,528	rs739048621	BT	Novel Gene	ENSGALG00000014643	G/A	0.947	0.052	0.894	212	0.105	25	0.000	0	0.7875	0.00
26	1,010,017	c.482C > T	ABF	*MYBPH*	ENSGALG00000000164	C/T	0.764	0.236	0.616	146	0.295	70	0.088	21	0.0071*	0.04
26	1,053,832	c.383C > T	ABF	*CEPT1*	ENSGALG00000000142	C/T	0.941	0.059	0.881	209	0.118	28	0.000	0	0.7402	0.02
26	1,086,300	rs312325687	ABF	Novel Gene	ENSGALG00000000104	A/G	0.639	0.361	0.443	105	0.392	93	0.164	39	0.0124	0.01
26	1,300,802	rs314560661	ABF	*AHCYL1*	ENSGALG00000000329	T/C	0.941	0.059	0.881	209	0.118	28	0.000	0	0.7875	0.01
26	1,312,073	rs14297872	ABF	*STRIP1*	ENSGALG00000037995	C/T	0.932	0.068	0.865	205	0.135	32	0.000	0	0.6067	0.01
26	1,510,415	rs741234441	ABF	Novel Gene	ENSGALG00000000477	C/T	0.605	0.395	0.320	76	0.569	135	0.109	26	2.101E-6*	0.01
26	1,779,214	rs733369312	ABF	*PPP1R15B*	ENSGALG00000000611	C/A	0.624	0.376	0.299	71	0.649	154	0.050	12	5.457E-13*	0.00
26	1,981,145	rs731705610	ABF	*CNTN2*	ENSGALG00000000653	C/T	0.947	0.053	0.894	212	0.105	25	0.000	0	0.9367	0.02
26	3,145,562	rs738655377	BT	Novel Gene	ENSGALG00000028858	A/G	0.863	0.137	0.725	172	0.274	65	0.000	0	1.0E-4*	0.00
26	3,213,394	rs736010549	BT	*WDR77*	ENSGALG00000040864	A/T	0.810	0.190	0.628	149	0.362	86	0.008	2	0.1768	0.01
26	3,290,417	rs737237434	BT	*DDX20*	ENSGALG00000001504	A/G	0.780	0.220	0.605	143	0.347	82	0.046	11	0.8671	0.01
26	3,747,346	rs14300225	BT	*PTPN22*	ENSGALG00000021656	C/T	0.084	0.916	0.004	1	0.160	38	0.835	198	0.7153	0.00
26	3,764,766	rs739340698	BT	*AP4B1*	ENSGALG00000035295	C/T	0.935	0.065	0.873	207	0.122	29	0.004	1	1.0	0.02
26	3,947,129	rs313532967	BT	*BARL*	ENSGALG00000002170	A/G	0.736	0.264	0.493	117	0.485	115	0.021	5	5.410E-5*	0.00
26	3,971,333	rs741234600	BT	*SYCP1*	ENSGALG00000002511	A/C	0.950	0.050	0.881	209	0.118	28	0.000	0	0.5256	0.03

*GGA, *Gallus gallus* chromosome; R, reference allele; and A, alternative allele; HREF, homozygous for the reference allele; HT, heterozygous; HALT, homozygous for the alternative allele; HW, Hardy-Weinberg; Freq., allele frequency; N, number of animals. SIFT, score predicted in functional annotation; ^∗^Significant *p*-value with Bonferroni multiple test correction (alpha 0.05). ^1^SNP ID from dbSNP the NCBI database. ^2^DR traits: DRW and DR%, BT traits: BTW and BT%, ABF traits: ABFW and ABF% TH traits THW and TH%.*

### Association Analysis for Additive and Dominance Models

We used both additive and non-additive models to verify if any of the deleterious SNP identified in each of the candidate genes were associated with the phenotype detected in the GWAS analysis. Detailed results for association tests (*p-values* and effects) between the SNPs and the eight traits are presented in Table 5 for the test for additive effects and Table 6 for the test for dominance. It is important to keep in mind that, for each trait, the association test was performed only with the SNPs located in the QTL region chosen for the respective trait. Based on the additive model, from the 18 predicted deleterious SNPs tested, c.482C > T was the only locus significantly associated with ABFW and ABF% (Table 5). In the analysis testing for dominance using a non-additive model, there were no SNPs with significant effects for any of the eight traits (Table 6).

**TABLE 5:** Association analysis results, *p-values* (upper value in cell) and effects (lower value in cell) for an additive model applied to carcass traits in the TT Reference population.

**SNP ID**	**BTW**	**BT%**	**THW**	**TH%**	**DRW**	**DR%**	**ABFW**	**ABF%**
rs739508259					0.9304	0.8735		
					−0.11g	−0.01%		
rs314536739			0.5326	0.3721				
			−1.45g	−0.10%				
rs739048621	0.5021	0.5647						
	−3.91g	0.16%						
c.482C > T							0.0474*	0.0332*
							−3.43g	−0.17%
c.383C > T							0.8783	0.5220
							0.44g	−0.08%
rs312325687							0.2750	0.1087
							−1.80g	−0.12%
rs314560661							0.6397	0.5073
							−1.33g	−0.09%
rs14297872							0.5599	0.2315
							1.74g	0.16%
rs741234441							0.6627	0.6188
							−0.79g	−0.04%
rs733369312							0.4361	0.2519
							−1.34g	−0.09%
rs731705610							0.1579 −4.50g	0.3106 −0.15%
rs738655377	0.0921	0.0993						
	−6.68g	0.31%						
rs736010549	0.2010	0.2112						
	−5.06g	0.24%						
rs737237434	0.1571	0.2225						
	−4.32g	0.18%						
rs14300225	0.8481	0.9848						
	0.92g	0.01%						
rs739340698	0.7328	0.6122						
	1.75g	0.12%						
rs313532967	0.0915	0.1617						
	5.55g	0.22%						
rs741234600	0.3910	0.4561						
	4.53g	0.18%						

*^∗^Significant at *p-value* < 0.05. BTW, breast weight; BT%, breast yield; THW, thigh weight; TH%, thigh yield; DRW, drumstick weight; DR%, drumstick yield; ABFW, abdominal fat weight; ABF%, abdominal fat yield. Effect values (bottom number) are presented in grams (g) for weight traits and percentage (%) for yield traits. Empty spaces demonstrate that no association test between SNP and phenotype was performed, since we only tested deleterious SNPs with their respective phenotype discovered in the GWAS analysis.*

**TABLE 6:** Association analysis results (*p-values*) for dominance effects based on a non-additive model applied to carcass traits.

**SNP ID**			**BTW**	**BT%**	**THW**	**TH%**	**DRW**	**DR%**	**ABFW**	**ABF%**
rs739508259	*p-value*						0.7921	0.9865		
	GE	1					−1.28g	−0.01%		
		2					−1.46g	−0.02%		
rs314536739	*p-value*				0.5032	0.5591				
	GE	1			−3.55g	−0.14%				
		2			−0.58g	−0.04%				
rs739048621	*p-value*		0.9651	0.9377						
	GE	1	−0.29g	0.02%						
c.482C > T	*p-value*								0.4367	0.4348
	GE	1							−1.36g	−0.04%
		2							−4.24g	−0.19%
c.383C > T	*p-value*								0.2185	0.5600
	GE	1							−1.52g	−0.14%
rs312325687	*p-value*								0.4817	0.8069
	GE	1							−1.14g	−0.08%
		2							−1.90g	−0.16%
rs314560661	*p-value*								0.9648	0.5887
	GE	1							1.41g	−0.01%
rs14297872	p-value								0.2156	0.6071
	GE	1							1.40g	0.15%
rs741234441	*p-value*								0.8325	0.7206
	GE	1							0.27g	0.05%
		2							2.64g	0.0%
rs733369312	*p-value*								0.3490	0.6812
	GE	1							0.42g	0.02%
		2							−2.79g	−0.22%
rs731705610	*p-value*								0.1669	0.1169
	GE	1							−4.68g	−0.19%
		2								
rs738655377	*p-value*		0.6615	0.0929						
	GE	1	−1.97g	−0.30%						
rs736010549	*p-value*		0.1221	0.1850						
	GE	1	−8.18g	−0.31%						
		2	16.66g	0.48%						
rs737237434	*p-value*		0.5131	0.4639						
	GE	1	−2.69g	−0.08%						
		2	−10.04g	−0.45%						
rs14300225	*p-value*		0.9423	0.8744						
	GE	1	−9.57g	−0.46%						
		2	−9.98g	−0.52%						
rs739340698	*p-value*		0.9463	0.6662						
	GE	1	1.43g	0.22%						
		2	−6.63g	−0.25%						
rs313532967	*p-value*		0.6065	0.1715						
	GE	1	3.88g	0.29%						
		2	−1.34g	−0.08%						
rs741234600	*p-value*		0.2294	0.1994						
	GE	1	7.20g	0.30%						

*GE, Genotype Effect; value 1 refers to heterozygotic allele for the reference and value 2 refers to homozygotic allele for the reference for each SNP. BTW, breast weight; BT%, breast yield; THW, thigh weight; TH%, thigh yield; DRW, drumstick weight; DR%, drumstick yield; ABFW, abdominal fat weight; ABF%, abdominal fat yield. Effect values (bottom number) are presented in grams (g) for weight traits and percentage (%) for yield traits. Empty spaces demonstrate that no association test between SNP and phenotype was performed.*

The SNP c.482C > T is in the Myosin Binding Protein H (*MYBPH*) gene and within the QTL region for ABFW and ABF% traits in GGA26. This polymorphism is a C > T change, resulting in the amino acid change of threonine to methionine at amino acid position 161 (T161M). In the additive model, the effects of the tested predicted deleterious SNP c.482C > T were −0.17% and −3.43g for ABF% and ABFW traits, respectively. Functional annotation analysis performed in Amigo 2 GO of this gene reported gene ontology (GO) terms related to protein binding (GO:0005515), regulation of striated muscle contraction (GO: 0006942), cell adhesion (GO:0007155), structural constituent of muscle (GO:0008307) and myosin filament (GO:0032982).

## Discussion

The economic importance of carcass weight and yield motivated the search for genomic regions and candidate genes associated with these traits (Coutinho et al., 2010; Boschiero et al., 2013; Van Goor et al., 2015; Derks et al., 2018). Combining functional studies and association analyses helped us to focus on positionally supported polymorphisms with the main goal of finding putative causal mutations for carcass traits. Here we present a report with genomic association analysis combining information of genomic regions and whole genome sequence to support the selection of predicted deleterious SNPs for association analysis with carcass traits in chickens.

From GWAS analysis, 19 unique QTL for THW, TH%, DRW, DR%, BTW and BT%, were detected, cumulatively explaining 0.54, 1.19, 8.54, 8.11, 2.64, and 4.97% of the genetic variance, respectively, with PPA > 0.69. These QTL are useful for further applications such as positional candidate genes search, gene network analysis and discovery of putative candidate mutations, as we investigated herein.

From the 19 QTL identified, five genomic windows were used here (four identified in this study and one characterized by Moreira et al., 2018b). Whitin these regions, we selected SNPs that had SIFT scores ranging from 0.0 to 0.04. Lower scores represents higher confidence and better sensitivity for detecting deleterious SNPs (Ng and Henikoff, 2003). All SNPs had an amino acid change prediction considered as moderate impact; no substitution resulted in a premature stop codon.

Deviation from HWE is an indication that allele frequency is not consistent with genotype frequency. Several evolutionary forces can affect HWE and a deleterious SNP could drive a deviation from HWE. Four predicted deleterious polymorphisms exhibited significant departures from HWE (*p-value* < 0.05 after bonferroni correction for multiple testing). Deviations from HWE may suggest that selection has affected the allele frequency.

It is pertinent to note that even though rs738655377 was not significantly associated with BTW or BT%, none of the animals genotyped were homozygous for the alternative allele, and the test for departure from HWE was significant. This variant is within a novel gene (ENSGALG00000028858) that presents gene ontology terms related to oxidoreductase activity. Out of the 237 animals genotyped, the expectation based on the observed allele frequency was that 4.45 animals would be homozygous for the alternative allele. The lack of homozygous animals for the alternative allele provides suggestive evidence of a lethal polymorphism when in homozygosity. However, further genotyping studies should be performed to confirm these findings based on matings between heterozygotes. Previous studies in cattle and pigs have identified lethal mutation by searching for lack of homozygous alleles (VanRaden et al., 2011; Kuehn et al., 2013; Derks et al., 2017; Jenko et al., 2019).

The significantly associated polymorphism, c.482C > T, located in the *MYBPH* gene is a novel SNP that caused a threonine-to-methionine substitution at amino acid position 161 (T161M). Threonine amino acid is polar (uncharged), while methionine is a non-polar amino acid. In Myosin-binding protein H, the substitution is located in the Fibronectin type-II domain (UniProt accession number Q05623 and PROSITE accession number PS50853) and this domain is the most common of the fibronectin subdomains (Hynes, 1990). Fibronectin is a dimeric glycoprotein involved in cell adhesion, cell morphology, cell migration, and embryonic differentiation.

The *MYBPH* presents gene ontology terms related to muscle growth and development such as: protein binding, regulation of striated muscle contraction, cell adhesion, structural constituent of muscle and myosin filament. In chickens, an increase in the amount of MYBPH protein in breast muscle is associated with muscle development at 56 days of age (Liu et al., 2016). In cattle, the *MYBPH* gene was previously shown to be associated with intramuscular fat deposition (Poleti et al., 2018; Bazile et al., 2019). A proteomic study performed in *Longissimus dorsi* samples in cattle found that MYBPH protein was significantly more abundant (*p-value* < 0.05) in a group of cattle exhibiting low intramuscular fat content (Poleti et al., 2018). Another bovine proteomic study found a significantly lower abundance of MYBPH protein in the high adiposity group (Bazile et al., 2019). Other studies with Nellore cattle found *MYBPH* gene differentially expressed when comparing groups with high and low intramuscular fat content (Cesar et al., 2015; dos Santos Silva et al., 2019). In chickens, the MYBPH was expressed in skeletal muscle tissue, heart, spleen, brain, kidney and female gonad (UniProt Databse, Bateman et al., 2021). Based on the association results in our population, the allele substitution effect of the predicted deleterious SNP was a reduction in 3.43 g of ABWF and 0.17% of ABF% for each copy of the alternative allele in the genotype.

It is important to mention that the polymorphism c.482C > T herein might not be the causal mutation because it was in strong linkage disequilibrium (LD block GGA26 1008728 Mb – 1015596 Mb with 0.92 r^2^) with two other polymorphisms in the *MYBPH* gene, rs313948592 (A/C, located in the intron) and rs312491203 (G/A change, located upstream of the gene). We are aware that polymorphism in intron or upstream regions can have regulatory impact and we cannot rule predicted deleterious SNP as causative mutations (Jo and Choi, 2015; Li et al., 2020). Nevertheless, this study indicates that MYBPH gene could have an impact on fat deposition in chickens. Further functional studies may help to elucidate the role of *MYBPH* and its polymorphisms in regulating fatness in meat-type chickens. Also, the other polymorphisms identified in the target regions are available at European Variation Archive (EVA) – EMBL-EBI (accession PRJEB25004) for further studies of causal mutations in these regions.

Our strategy has provided novel evidence in the identification of a putative causative mutation associated with abdominal fatness in meat-type chickens. We report one predicted deleterious SNP associated with abdominal fat traits located in the *MYBPH* gene that is a candidate gene for fat deposition. The main limitation of our study is not being able to discriminate whether the identified mutation is the causative mutation, or it is simply in strong linkage disequilibrium with the real causal mutation. Nevertheless, our findings can be applied in poultry breeding programs focused on carcass traits improvement.

## Data Availability Statement

All data generated or analyzed during this study are public and included in this published article. The SNP report (identified by sequencing) was submitted to European Variation Archive (EVA) – EMBL-EBI, accession number PRJEB25004. The datasets used and/or analyzed during the current study (genotypes and phenotypes) are available from the corresponding author on reasonable request.

## Ethics Statement

In this study, all experimental protocols that used animals were performed in agreement with resolution number 010/2010 approved by the Embrapa Swine and Poultry Ethics Committee on Animal Utilization (CEUA) in Concordia, Santa Catarina State – South of Brazil, in agreement with the rules of National Council of Animal Experimentation Control (CONCEA) to ensure compliance with international guidelines for animal welfare.

## Author Contributions

PT, GCM, CB, ML, GBM, and LC conceived the idea of this research and participated in the experimental design. ML and LC provided the experimental environment, phenotype, and data analysis support. PT, GCM, CB, JP, AC, GRM, and DG performed data analysis. PT drafted the manuscript. PT, GCM, CB, AC, JP, GRM, ML, GBM, DG, and LC collaborated with interpretation, discussion, and writing of the manuscript. All authors have read and approved the final manuscript.

## Conflict of Interest

FAPESP, CAPES, CNPq, and the Brazilian Government through Embrapa provided financial support to generate the data; however, they did not participate in the design of the study, sample collection, analysis, data interpretation, and writing of the manuscript. The authors declare that the research was conducted in the absence of any commercial or financial relationships that could be construed as apotential conflict of interest.

## Publisher’s Note

All claims expressed in this article are solely those of the authors and do not necessarily represent those of their affiliated organizations, or those of the publisher, the editors and the reviewers. Any product that may be evaluated in this article, or claim that may be made by its manufacturer, is not guaranteed or endorsed by the publisher.

## Footnotes

1
https://www.ebi.ac.uk/ena/data/view/PRJEB25004

2
http://amigo.geneontology.org

3
http://www.r-project.org/

## Supplementary Material

Supplementary Table 1 Characterization of all the 947 SNP windows analyzed.
